# No effects of Korean pine nut triacylglycerol on satiety and energy intake

**DOI:** 10.1186/1743-7075-8-79

**Published:** 2011-11-10

**Authors:** Sanne PM Verhoef, Klaas R Westerterp

**Affiliations:** 1 Maastricht University, Department of Human Biology, Nutrition and Toxicology Research Institute Maastricht, 6200 MD, Maastricht, The Netherlands

**Keywords:** Visual analogue scale, triacylglycerol, appetite suppressant, PinnoThin

## Abstract

**Background:**

Triacylglycerols (TAG) have been shown to have potential appetite suppressing effects. This study examined the effects of 3 g and 6 g Korean pine nut triacylglycerols (PinnoThin) on appetite and energy intake.

**Methods:**

130 g Isoenergetic yogurt containing either placebo (milk fat) or PinnoThin TAG was consumed as a breakfast, after an overnight fast, in a double blind randomized crossover design. Appetite profile ratings were determined by visual analogue scale at regular intervals for a period of 4 h after the breakfast. In phase I, 6 g PinnoThin TAG and placebo was tested in thirty-three healthy women (mean ± SD, BMI 26.4 ± 3.8 kg/m^2^; age 28 ± 10 y) to determine the appetite suppressing effect in time. In phase II, an additional dose of 3 g PinnoThin TAG, as well as 6 g PinnoThin TAG and placebo, was tested in thirty-four women (BMI 25.8 ± 2.9 kg/m^2^; age 25 ± 9 y) to determine energy intake from an *ad libitum *lunch offered at 210 min after the breakfast, at which maximal differences in appetite profile ratings were present in phase I.

**Results:**

Area under the curve of appetite profile ratings was not significantly different between the conditions. Energy intake was 9.5% lower after 6 g PinnoThin TAG compared with 3 g PinnoThin TAG, but there was no significant difference with the placebo.

**Conclusion:**

A dosage of 6 g PinnoThin TAG is not sufficient to suppress appetite and energy intake.

**Trial registration:**

Clinical Trials NCT01034605

## Introduction

An effective strategy to prevent the positive energy balance resulting in obesity is still lacking. It has been shown that a positive energy balance of 50 kcal per day already results in an increase in body weight of at least one kilo per year [1]. A potential prevention strategy is by reducing food intake via the use of (natural) appetite suppressants.

Different macronutrients exert various postprandial effects and stimulate the release of satiety hormones to different degrees. Fat intake is known to induce the release of gastro-intestinal hormones, like cholecystokinin (CCK) and glucagon-like peptide-1 (GLP-1). Both CCK and GLP-1 have shown to be involved in the development of satiation, by suppressing appetite and energy intake [2-5]. The satiating effect of fat is influenced by chain length and the degree of saturation of the fatty acids. Only fatty acids with chain lengths ≥ C12 are capable of reducing food intake and releasing CCK and GLP-1 [6,7]. Furthermore, long-chain fatty acids have been shown to be more effective than medium-chain fatty acids [8,9]. Regarding the degree of saturation, it has been shown that polyunsaturated fatty acids (PUFAs) have a more potent effect on reducing food intake and increasing the release of satiety hormones than monounsaturated fatty acids (MUFAs) [8].

PinnoThin is an oil from Korean pine nuts (*Pinus Koraiensis*) which consists of 66% polyunsaturated fatty acids (PUFAs; linoleic (C18:2) and pinolenic acid (C18:3)) and 26% monounsaturated fatty acids (MUFAs; oleic acid (C18:1)) [10]. The large amounts of PUFAs in PinnoThin suggest that PinnoThin might be able to reduce appetite by the induction of satiety hormones.

Previous studies have shown that PinnoThin triacylglycerol (TAG) and free fatty acids (FFA) can produce an increase in CCK and GLP-1 release in post-menopausal overweight women [11]. AUC of appetite ratings were not significantly different after the consumption of PinnoThin (FFA and TAG) [11], probably because the study was underpowered with only 18 overweight postmenopausal women. Moreover, observations were not corrected for baseline measurements, leading to the question whether the results were based upon clear statistically significant differences between treatment and placebo. No effects of PinnoThin TAG on appetite ratings were found in a study with 42 overweight women [12]. In contrast, in the same study 2 g PinnoThin FFA resulted in a trend for reduced energy intake (7% reduction, P = 0.09) [12]. The authors suggest that the lack of an effect of PinnoThin TAG could be attributed to the timing of the dosing regime. Absorption of fatty acids in the gut from TAG is slower compared to FFA, consequently the time between consumption of PinnoThin TAG and the *ad libitum *lunch (30 min) was probably too short to detect an effect.

The present study was designed to measure the effect of PinnoThin TAG (66% PUFAs and 26% MUFAs), in a sufficient number of subjects. In a first phase, the effect of PinnoThin TAG on appetite ratings was evaluated. To overcome the problem of timing of the dosing regime we determined the sensitive moment in time, at which maximal differences between one dosage of PinnoThin TAG and placebo in appetite profile ratings were present, in this first phase. In a second phase, the effect of two dosages of PinnoThin TAG vs. placebo on energy intake at this previously determined relevant time point was evaluated to determine the minimum effective dosage.

## Subjects and methods

### Subjects

Thirty-nine healthy women aged 18-45 y with a BMI of 23-30 kg/m^2 ^were recruited by advertisements in local newspapers and on notice boards at the university. All subjects underwent a screening and were in good health, regularly consuming breakfast, nonsmokers, not using medication (except for oral contraception) and at most moderate alcohol users. None of the subjects had a food allergy, had gained or lost more than 5 kg in three months prior to the study, or were cognitively dietary restrained (F1 > 13) as assessed by a validated Dutch translation of the Three Factor Eating Questionnaire (TFEQ) [13]. This study was conducted according to the guidelines laid down in the Declaration of Helsinki and all procedures involving human subjects were approved by the Central Committee on Human Research and by the Medical Ethical Committee of the University of Maastricht. Written informed consent was obtained from all subjects.

### Study protocol

The study had a double blind, randomized, crossover design. Test day were scheduled in the same phase of the subjects' menstrual cycle, at least one menstrual cycle apart. Subjects were provided with a meal to consume at home on the evening prior to each test day. They were asked to abstain from strenuous physical activity and not to eat or drink from 2200 h the night before the test day. The subjects reported to the university at 0845 h after an overnight fast. At 0900 h the subjects received a yogurt breakfast (130 g) containing either PinnoThin TAG or placebo (milk fat).

The study consisted of two phases, with the results of phase I determining the timing of the measurement in phase II. During phase I, appetite ratings were determined at regular intervals for a period of 4 h after the test breakfast with 6 g PinnoThin TAG or 6 g placebo. In phase II, an *ad libitum *lunch was offered at the previously determined sensitive moment in time, based on the maximal differences in appetite profile ratings in phase I. In addition to 6 g PinnoThin TAG and 6 g placebo, an extra dose of 3 g PinnoThin TAG was tested with a yogurt breakfast containing a mixture of 3 g PinnoThin TAG and 3 g milk fat.

### Appetite ratings

Appetite was evaluated using anchored 100-mm visual analogue scales (VAS) in both phase I and II [14,15]. Hunger, fullness, satiety, thirst, desire to eat, prospective food consumption and nausea were assessed. The scale was anchored from 'not at all' on the left to 'extremely' on the right. On each test day, these questionnaires were completed before breakfast at 0855 h, after breakfast at 0915 and 0930 h and every 30 minutes thereafter (1000, 1030, 1100, 1130, 1200, 1230 and 1300 h).

### Yogurt breakfast

Breakfast was a yogurt (130 g) containing either 6 g milk fat, 3 g PinnoThin TAG and 3 g milk fat or 6 g PinnoThin TAG, which provided 46, 11 and 43 En% from carbohydrate, protein and fat respectively. The energy content of the breakfast was 523kJ. PinnoThin TAG consists of 66% polyunsaturated fatty acids (PUFAs; linoleic (C18:2) and pinolenic acid (C18:3)) and 26% monounsaturated fatty acids (MUFAs). The subjects were instructed to consume the yogurt within 5 minutes and to hold each spoon of yogurt in their mouth for 5-10 seconds before swallowing.

The yogurts were produced by NIZO Food Research b.v. (Ede, The Netherlands) with either PinnoThin TAG (Lipid Nutrition b.v., Wormerveer, The Netherlands) or milk fat (Corman b.v., Goé, Belgium) and were flavored with a mixture of orange, apple (SVZ, Breda, The Netherlands), lemon and passion fruit (Givaudan b.v., Barneveld, The Netherlands). The yogurts did not differ in color, flavour or viscosity.

### Energy intake

Food and energy intake were determined using an *ad libitum *lunch in phase II. Lunch consisted of Turkish bread (330 g) with egg salad (600 g) and was weighed before and after eating. Subjects were instructed to eat till they were comfortably full. The lunch had an energy density of 11.5kJ/g with 44, 17 and 39 En% from carbohydrate, protein and fat respectively.

### Statistical analysis

Since baseline values did not differ, data are presented as mean changes from baseline and their standard errors, unless otherwise indicated. The area under the curve (AUC) of changes from baseline over time (4 h for appetite ratings) was calculated using the trapezoid method. A repeated-measures ANOVA was carried out to determine possible differences in appetite ratings and energy intake between the PinnoThin TAG (3 g and 6 g) and placebo breakfast. Significance was defined as *P *< 0.05, unless otherwise indicated. A power calculation was performed to determine the number of subjects required and was based on a difference in food intake after intake of 2 g PinnoThin FFA observed in a previous study [12]. With an observed difference of 7% and a standard deviation of 10%, it was calculated that after taking a 15% dropout into account 30 subjects were needed to achieve sufficient power (90%) to observe significant (P < 0.05) changes in food intake as a result of the treatment. All of the statistical analyses were executed with SPSS version 16.0 for Macintosh OS X (SPSS Inc, Chicago, IL).

## Results

### Subject characteristics

Thirty-three women participated in phase I. Five women of this group did not participate in phase II, because of personal reasons and/or lack of time. Six new female subjects were recruited for phase II, and together with the remaining 28, a total of 34 women completed the second set of experiments. Subject characteristics of both phases are presented in table 1.

**Table 1:** Subject characteristics

	Phase I		Phase II	
	33 women		34 women*	
	Mean	SEM	Mean	SEM
Age (y)	28	2	25	2
Height (m)	1.69	0.01	1.70	0.01
Body Weight (kg)	75.6	1.9	74.3	1.7
BMI (kg/m^2^)	26.4	0.4	25.8	0.5
Dietary restraint^‡^	7.1	0.7	6.5	0.7

Values are expressed as mean ± SEM. BMI = Body Mass Index

*28 subjects completed both phases. Five women dropped out before phase II was completed and therefore six new subjects were recruited for phase II.

^‡^Dietary restraint = Factor 1 score of the Three-Factor Eating Questionnaire [13]

### Phase I

**Figure 1:**
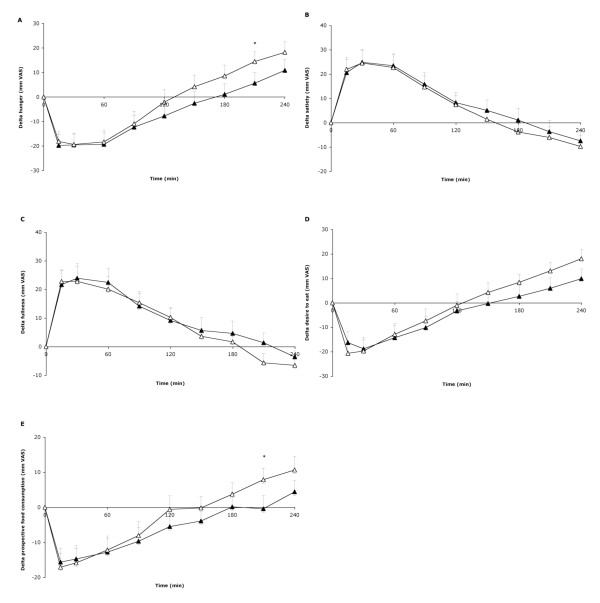
**Changes in hunger (A), satiety (B), fullness (C), desire to eat (D) and prospective food consumption (E) (all in mm visual analogue scale; VAS) over time (min) after a yogurt breakfast with either placebo (△) or 6 g PinnoThin TAG (▲) expressed as changes from baseline in 33 women (phase I)**. Values are means, with standard errors represented by vertical bars. *P < 0.05 with repeated-measures ANOVA.

Hunger ratings were significantly decreased after 6 g PinnoThin TAG compared with placebo at 210 min after the breakfast (6 (SEM 4) v. 14 (SEM 4) mm VAS; P < 0.05; Figure 1a). Prospective food consumption was decreased after PinnoThin TAG compared to placebo at 210 min after the breakfast (0 (SEM 4) v. 8 (SEM 3) mm VAS; P < 0.05; Figure 1e). The time-by-treatment interaction did not reach significance and the AUC for the appetite ratings was not significantly different between the treatments. Based on these results, in phase II an *ad libitum *lunch was offered at 210 min after breakfast with placebo, 3 g or 6 g PinnoThin TAG.

### Phase II

The time-by-treatment interaction did not reach significance and also the AUC for the appetite ratings was not significantly different between the treatments. Appetite ratings over time were comparable between phase I and II.

Energy intake was decreased significantly by 9.5% after the breakfast with 6 g PinnoThin TAG compared with the breakfast with 3 g PinnoThin TAG (P < 0.05; Table 2). The lowered energy intake after 6 g PinnoThin TAG did not reach significance compared with the placebo. In addition, there was no significant difference between energy intake after 3 g PinnoThin TAG and placebo. These results remain similar after correcting energy intake for body weight, height and age of each subject. This was done by defining energy intake as a % of energy requirement (ER), calculated according to the Harris-Benedict equations, multiplied by an activity index of 1.7 [16].

**Table 2:** Food and energy intake (phase II)

	Placebo		3 g PinnoThin TAG		6 g PinnoThin TAG	
	Mean	SEM	Mean	SEM	Mean	SEM
Food intake (gram)	312	13	327	11	296	11*
Energy intake (kJ)	3594	150	3766	134	3409	130*

Values are expressed as mean ± SEM. Food (gram) and energy (kJ) were measured with an *ad libitum *lunch 210 min after a breakfast with placebo, 3 g and 6 g PinnoThin TAG in 34 women. *Significant different from 3 g PinnoThin: P < 0.05 (repeated-measures ANOVA).

Ratings of liking of the yogurt breakfast, nausea and thirst were not different between the treatments. Nausea ratings were extremely low and changes from baseline over time did not become positive. No adverse events were reported.

## Discussion

The time-by-treatment interaction and differences in AUC for the appetite ratings were not significant in both phases. Energy intake was reduced by 9.5% after supplementation of 6 g PinnoThin TAG compared with 3 g PinnoThin TAG. However, the reduction in energy intake after 6 g PinnoThin TAG when compared with placebo did not reach significance.

If PinnoThin TAG indeed acts as an appetite suppressant, it would be expected that PinnoThin TAG suppresses appetite measured via appetite profile ratings with VAS. However, the time-by-treatment interaction was not significant. In the study of Hughes et al. [12], there were also no effects of 6 g PinnoThin TAG on appetite ratings, and in the study of Pasman et al. [11] PinnoThin TAG only marginally affected appetite ratings. These studies had major differences between the study protocols. For example, PinnoThin was consumed 3.5 h after the breakfast in the Hughes study, instead of simultaneously with a breakfast after an overnight fast in this study, as well as the Pasman study. Previous studies supplemented PinnoThin in a capsule [11,12], whereas in this study PinnoThin TAG was incorporated in a yogurt breakfast. Also, milk fat instead of olive oil was used as a placebo in this study. In addition, the Pasman study was underpowered with only 18 postmenopausal overweight women [11]. Our sample size was sufficiently large to reach power.

Differences between FFA and TAG also cause the contradictory results in the literature. FFAs have a stronger effect on CCK release, fullness and hunger ratings and energy intake compared with TAGs [17]. FFAs can directly exert effects in the gut, whereas FFAs from TAGs are released during intestinal lipolysis. In the Hughes study food intake (grams), and not energy intake (kJ), was reduced during an *ad libitum *lunch 30 min after supplementation of 2 g FFA, while PinnoThin TAG did not have an effect on food nor energy intake [12]. It has been suggested that the short amount of time between PinnoThin TAG supplementation and the *ad libitum *lunch can explain the absence of an effect on energy intake in Hughes study [12]. The rate of small intestinal lipolysis of TAGs is unknown, and seems to depend on several factors [17], like the rate of pancreatic lipase secretion and also the rate of entry of TAGs into the duodenum. In turn, the latter depends on the rate of gastric emptying, which varies with the amount and physical properties of the meal [18]. Therefore, the concentration of FFA required in the intestine to suppress energy intake is still unclear [17]. Furthermore, only two out of three fatty acids will become available from TAG. This suggests that a same dosage of FFA would elicit a stronger effect on energy intake compared with TAG. To get around these unknown variables we optimized the measurements by determining energy intake at a previous determined sensitive moment in time, at which maximal differences in appetite profile ratings were present. However, the reduction in energy intake after 6 g PinnoThin TAG when compared with placebo did not reach significance. The observed effects of PinnoThin TAG may become stronger when a higher dose is supplemented. However, a higher dose is not desirable for physiological and industrial reasons. With PinnoThin TAG being a fat, a higher dose might not result in a net reduction of total daily fat intake. Furthermore, the acute effects of a single dose of PinnoThin TAG could be different from the effects after a long-term trial, but no studies thus far have investigated the effect of PinnoThin TAG in the long term.

In this study, unlike earlier studies, the effect of 2 dosages PinnoThin TAG on energy intake is determined at a previously determined sensitive moment in time, in a sufficiently large number of subjects to reach power (to detect an effect size of 7% with a power of 90% at P = 0.05). Although no appetite suppressing effect of PinnoThin TAG is found in the concentrations used in this study, the results significantly contribute to the field of research on potential appetite suppressants. Clearly, a lowest dosage as possible is preferred for physiological as well as industrial reasons. In order to find a minimum effective dosage it is important to perform studies with several dosages. Further studies need to be done to determine whether higher concentrations of PinnoThin TAG do have an appetite suppressing effect. From these results we conclude that a dosage of 6 g PinnoThin TAG is not sufficient to suppress appetite sensations and energy intake.

## Competing interests

The authors declare that they have no competing interests.

## Authors' contributions

SV collected and analyzed the data and wrote the manuscript. KW contributed to the interpretation of the data and reviewed the manuscript. The study was executed under supervision of KW. All authors read and approved the final manuscript.
